# Supplementary material for: Association of intra-articular injection and knee arthroscopy prior to primary knee replacement with the timing and outcomes of surgery: Retrospective cohort study using data from the Clinical Practice Research Datalink GOLD database
Source: PLoS One. 2024 Nov 21;19(11):e0311947. doi: 10.1371/journal.pone.0311947 (PMC11581240; doi:10.1371/journal.pone.0311947)
Supplement: S1 Table — (DOCX) [file pone.0311947.s001.docx]

***S1 Table. Table displaying the number of patients within our study population that underwent both Intra-articular Steroid Injection (IASI) and Knee Arthroscopy (KA) prior to primary knee replacement.***

| **Prior KA** | **Prior IASI** | |  |
| --- | --- | --- | --- |
|  | No | Yes | Total |
| No | 21,314 | 10,205 | 31,519 |
| Yes | 4,040 | 2,307 | 6,347 |
| Missing | 437 | 191 | 628 |
| Total | 25,791 | 12,703 | 38,494 |
