# Supplementary material for: Association of intra-articular injection and knee arthroscopy prior to primary knee replacement with the timing and outcomes of surgery: Retrospective cohort study using data from the Clinical Practice Research Datalink GOLD database
Source: PLoS One. 2024 Nov 21;19(11):e0311947. doi: 10.1371/journal.pone.0311947 (PMC11581240; doi:10.1371/journal.pone.0311947)
Supplement: S2 Table — (DOCX) [file pone.0311947.s002.docx]

***S2 Table. Characteristics of patients in the full CPRD versus CPRD-HES linked datasets***

|  | **CPRD dataset** | **CPRD-HES linked** |
| --- | --- | --- |
| **Confounder** | n=63899 | n=38494 |
|  | n (%) | n (%) |
| Male | 27758 (43.4%) | 16650 (43.3%) |
| Female | 36141 (56.6%) | 21844 (56.8%) |
| Age |  |  |
| mean (sd) | 69.2 (9.7) | 69.4 (9.7) |
| <60 | 10151 (15.9%) | 5938 (15.4%) |
| 60 to 70 | 21099 (33.0%) | 12566 (32.6%) |
| 70 to 79 | 23444 (36.7%) | 14288 (37.1%) |
| 80+ | 9205 (14.4%) | 5702 (14.8%) |
| BMI |  |  |
| Normal | 8404 (16.3%) | 5224 (16.8%) |
| Overweight | 19673 (38.1%) | 12049 (38.7%) |
| Obese Class I | 14612 (28.3%) | 8616 (27.7%) |
| Obese Class II | 6434 (12.5%) | 3794 (12.2%) |
| Obese Class III | 2518 (4.9%) | 1437 (4.6%) |
| Missing | 12,258 | 7,374 |
| Smoker |  |  |
| Ex | 19103 (34.1%) | 11662 (34.6%) |
| No | 31875 (56.9%) | 19064 (56.5%) |
| Yes | 5072 (9.1%) | 3024 (9.0%) |
| Missing | 12,258 | 4,744 |
| Alcohol Consumption |  |  |
| Ex | 1291 (2.8%) | 776 (2.8%) |
| No | 8379 (18.2%) | 4973 (17.9%) |
| Yes | 36301 (79.0%) | 21976 (79.3%) |
| Missing | 17,928 | 10,769 |
| Deprivation Index Rank |  |  |
| Least | 9928 (25.1%) | 9473 (24.6%) |
| 2 | 9481 (24.0%) | 9222 (24.0%) |
| 3 | 8690 (22.0%) | 8480 (22.1%) |
| 4 | 6608 (16.7%) | 6522 (17.0%) |
| Most | 4805 (12.2%) | 4759 (12.4%) |
| Missing | 24,387 | 38 |
| Charlson Score |  |  |
| None | 45245 (70.8%) | 27168 (70.6%) |
| 1 | 6494 (10.2%) | 3962 (10.3%) |
| 2 | 6717 (10.5%) | 4096 (10.6%) |
| 3 | 2337 (3.7%) | 1408 (3.7%) |
| 4+ | 3106 (4.9%) | 1860 (4.8%) |
