# Supplementary material for: Association of intra-articular injection and knee arthroscopy prior to primary knee replacement with the timing and outcomes of surgery: Retrospective cohort study using data from the Clinical Practice Research Datalink GOLD database
Source: PLoS One. 2024 Nov 21;19(11):e0311947. doi: 10.1371/journal.pone.0311947 (PMC11581240; doi:10.1371/journal.pone.0311947)
Supplement: Attachment — Submitted filename: Response to Reviewers.docx [file pone.0311947.s003.docx]

**Editors comments:**

**1. When submitting your revision, we need you to address these additional requirements.**

**Please ensure that your manuscript meets PLOS ONE's style requirements, including those for file naming. The PLOS ONE style templates can be found at https://journals.plos.org/plosone/s/file?id=wjVg/PLOSOne_formatting_sample_main_body.pdf and https://journals.plos.org/plosone/s/file?id=ba62/PLOSOne_formatting_sample_title_authors_affiliations.pdf**

We have ensured that the manuscript meets the journals style requirements.

**2. Please provide additional details regarding participant consent. In the ethics statement in the Methods and online submission information, please ensure that you have specified what type you obtained (for instance, written or verbal, and if verbal, how it was documented and witnessed). If your study included minors, state whether you obtained consent from parents or guardians. If the need for consent was waived by the ethics committee, please include this information.**

**Once you have amended this/these statement(s) in the Methods section of the manuscript, please add the same text to the “Ethics Statement” field of the submission form (via “Edit Submission”).**

**For additional information about PLOS ONE ethical requirements for human subjects research, please refer to http://journals.plos.org/plosone/s/submission-guidelines#loc-human-subjects-research.**

Details regarding participant consent has already been included within the manuscript under the heading ‘Ethical approval and informed consent’. This is a retrospective study of routinely collected primary care data from the Clinical Practice Research Datalink (CPRD) where data were already fully anonymised before being accessed for this study. CPRD has ethics approval from the Health Research Authority to support research using anonymised patient data. Researchers apply to CPRD for approval to access anonymised data for a research project. <https://www.cprd.com/safeguarding-patient-data>

The information below has been included within the manuscript and the same statement has been included to the ‘ethics statement’ field of the submission form:

*“No information that can identify a patient is ever sent to CPRD from the contributing GP practices. Because a patient can’t be identified from data a GP practice sends to CPRD, the GP practice doesn’t need to seek a patient’s consent to share data with CPRD (https://www.cprd.com/safeguarding-patient-data). CPRD has obtained ethical approval from a National Research Ethics Service Committee (NRES) for all purely observational research using anonymised CPRD data; namely, studies which do not include patient involvement. The study has been approved by ISAC (Independent Scientific Advisory Committee) for MHRA Database Research) (protocol number 17_127R). CPRD data linked to inpatient HES received on 9th May 2017; linked PROMs data on 25th July 2017. All data were fully anonymized before being accessed for this study by the authors. The authors had no access to information that could identify individual participants.”*

**3. Thank you for stating the following in the Competing Interests section:**

**All authors have completed the Unified Competing Interest form at www.icmje.org/coi_disclosure.pdf and declare: AJ reports personal fees from Freshfields Bruckhaus Derringer, personal fees from Anthera Pharmaceuticals Ltd, outside the submitted work; All other authors declare no conflicts of interest.**

**We note that one or more of the authors are employed by a commercial company.**

None of the authors are employed by a commercial company. Andrew Judge (AJ) has never been employed by a commercial company. AJ is currently a full time member of academic staff at the University of Bristol since September 2017 (previously full time academic staff member at the University of Oxford (May 2009 to September 2017)). During this period of time AJ conducted external consultancy work, that included payment of fees, as a member of the Data Safety and Monitoring Board (DSMB) for Anthera Pharmaceuticals Inc, San Francisco (13 Dec 2012 to 28 June 2016) and as an expert advisor for international law firm Freshfields Bruckhaus Deringer, London (11 Aug 2014 to 14 Feb 2018).

The competing interest statement has been amended to make it clear that this was external consultancy work.

This is all as previously described and declared in other recent Plos One publications by Andrew Judge e.g. <https://journals.plos.org/plosone/article?id=10.1371/journal.pone.0270274>

**a. Please provide an amended Funding Statement declaring this commercial affiliation, as well as a statement regarding the Role of Funders in your study. If the funding organization did not play a role in the study design, data collection and analysis, decision to publish, or preparation of the manuscript and only provided financial support in the form of authors' salaries and/or research materials, please review your statements relating to the author contributions, and ensure you have specifically and accurately indicated the role(s) that these authors had in your study. You can update author roles in the Author Contributions section of the online submission form.**

We have not amended the funding statement to declare a commercial affiliation, where as described above, AJ is not funded by a commercial organisation.

We confirm that existing author contributions statements are accurate.

**Please also include the following statement within your amended Funding Statement.**

**“The funder provided support in the form of salaries for authors, but did not have any additional role in the study design, data collection and analysis, decision to publish, or preparation of the manuscript. The specific roles of these authors are articulated in the ‘author contributions’ section.”**

**If your commercial affiliation did play a role in your study, please state and explain this role within your updated Funding Statement.**

We have amended the funding section to include the statement “The funder provided support in the form of salaries for authors AJ and MW, but did not have any additional role in the study design, data collection and analysis, decision to publish, or preparation of the manuscript. The specific roles of these authors are articulated in the ‘author contributions’ section.”

**b. Please also provide an updated Competing Interests Statement declaring this commercial affiliation along with any other relevant declarations relating to employment, consultancy, patents, products in development, or marketed products, etc.**

As described above, this is not a commercial affiliation. It is related to external consultancy work. The competing interest statement has been amended to be clear this is external consultancy.

**Within your Competing Interests Statement, please confirm that this commercial affiliation does not alter your adherence to all PLOS ONE policies on sharing data and materials by including the following statement: "****This does not alter our adherence to PLOS ONE policies on sharing data and materials.” (as detailed online in our guide for authors http://journals.plos.org/plosone/s/competing-interests) . If this adherence statement is not accurate and there are restrictions on sharing of data and/or materials, please state these. Please note that we cannot proceed with consideration of your article until this information has been declared.**

The statement has been added to the article, but this is not a commercial affiliation, as stated earlier.

**Please include both an updated Funding Statement and Competing Interests Statement in your cover letter. We will change the online submission form on your behalf.**

This has been clarified in the cover letter.

**4. Thank you for uploading your study's underlying data set. Unfortunately, the repository you have noted in your Data Availability statement does not qualify as an acceptable data repository according to PLOS's standards.**

**At this time, please upload the minimal data set necessary to replicate your study's findings to a stable, public repository (such as figshare or Dryad) and provide us with the relevant URLs, DOIs, or accession numbers that may be used to access these data. For a list of recommended repositories and additional information on PLOS standards for data deposition, please see** <https://journals.plos.org/plosone/s/recommended-repositories>**.**

This study used anonymised routinely collected primary care electronic data from the Clinical Practice Research Datalink (CPRD). We are not able to share the data used for this study, and access to CPRD data is subject to protocol approval via CPRD’s Research Data Governance (RDG) Process. We have amended the data sharing statement below:

*“Electronic health records are, by definition, considered sensitive data in the UK by the Data Protection Act and cannot be shared via public deposition because of information governance restriction in place to protect patient confidentiality. Access to data is available once approval has been obtained through the individual constituent entities controlling access to the data. The primary care data can be requested via application to the Clinical Practice Research Datalink, secondary care data can be requested via application to the Hospital Episode Statistics from the UK Health and Social Care Information Centre, and mortality data are available by application to the UK Office for National Statistics. Alternatively, access to secondary care data and mortality data is available through linkage requested upon application for primary care data to the Clinical Practice Research Datalink. Information regarding linkage is available from: https://www.cprd.com/linked-data (https://www.cprd.com/research-applications).”*

**5. Please include captions for your Supporting Information files at the end of your manuscript, and update any in-text citations to match accordingly. Please see our Supporting Information guidelines for more information: http://journals.plos.org/plosone/s/supporting-information.**

The captions for the supporting information files have been updated as requested including in-text citations as per the journal guidelines.

**Reviewer #1**

1. **In the introduction, the following references on treatment methods for osteoarthritis might be helpful to provide more information and benefit the future readers: e.g., Injectable hydrogel microspheres in cartilage repair, Biomedical Technology 2023, 1, 18-29./ Functional biomaterials for osteoarthritis treatment: From research to application. Smart Medicne 2022, e20220014./ Microenvironment-responsive nanosystems for osteoarthritis therapy. Engineered Regeneration 2024, 5, 92-110./ Emerging microfluidics for the modeling and treatment of arthritis. Engineered Regeneration 2024, 5, 153-169.**

The introduction has been updated to included further information on alternative injectable treatments in OA (PRP / biomaterials) and relevant guidance and citations included.

2. **The article mentions a three-fold increased risk of SSI within 3 months of IASI and knee replacement, but the event rate is low and difficult to interpret. It is suggested that the author further analyze this conclusion and discuss the reasons for the low rate of interpretation events and the influence on the statistical results.**

We identified an infection rate of 0.1% and absolute numbers of infections were low overall. As outlined in our limitations, we planned to capture infections in the same knee within 3 months of the intervention, however it is likely that some infections may not have been captured and recorded in the CPRD dataset. This may have underestimated the event rate and lead to type 1 errors.

The manuscript has been updated to reflect the above in the limitations section of the study. For interpretation of this finding in the discussion section, we highlight that caution is needed due to the low event rate, and that this may be a chance finding.

3. **“Both interventions were associated with a lower risk of 1-year mortality**

**following primary knee replacement, for which there is not a biologically plausible**

**mechanism, suggesting potential for selection bias due to confounding by indication**

**for patients receiving these interventions.” Please give a detailed discussion on why there is bias and what are the possible reasons?**

We believe that this is due to selection bias through confounding by indication whereby younger age at Primary Knee Replacement is the key factor in the KA group and is accounting for the reduced mortality.

We do not believe that there is a biological cause for the reduction in mortality seen with prior IASI users and provide possible explanations for the observed association of reduced risks of mortality with IASI and KA are outlined comprehensively further on in our discussion:

“It is possible the observed reduction in 1-year mortality associated with prior KA is the result of confounding by indication. Patients who had undergone prior KA where on average 5.7 years younger at the time of primary knee replacement than those that did not undergo prior KA (64.6 vs 70.3) (Table 1). Younger age at primary knee replacement has been associated with lower risk of mortality (28). It may also explain the higher revision rates seen in the KA group, younger patients at time of primary knee replacement are associated with increased life time risk of revision (28). Prior IASI was associated with reduced odds of 1-year mortality (OR=0.76 95%CI (0.58 to 1.00)), however much less so than prior KA, this may be due to more balanced demographics seen in prior IASI users and non-users, especially age (table 1). There is further potential for residual confounding that may be responsible for the reduction in OR for mortality in KA and IASI users. There may be important unknown and unmeasured confounders that where not accounted and controlled for in our analysis.”

4. **Is it possible to analyse patients who have received both intraarticular steroid injection and arthroscopic knee surgery as a category?**

| Prior KA | Prior IASI | |  |
| --- | --- | --- | --- |
|  | No | Yes | Total |
| No | 21,314 | 10,205 | 31,519 |
| Yes | 4,040 | 2,307 | 6,347 |
| Missing | 437 | 191 | 628 |
| Total | 25,791 | 12,703 | 38,494 |

The above table outlines the number of patients undergoing both IASI and KA which totals 2,307. This is 6% of our total patient population.

Our current research question for the paper, is whether or not a patient receiving prior IASI, or prior KA, is associated with timing and outcomes of surgery. Acknowledging that these are not mutually exclusive groups, and there will be some overlap of patients who have received both prior interventions. We have addressed this point in our discussion.

Whilst it would be possible to expand the paper and analyse the exposure as (prior IASI only, prior KA only, prior IASI and KA, no prior IASI and KA), numbers who have received both IASI and KA are small, and it would make the paper more complex expanding the tables, figures, and results section to include this further exposure group.

As such, our preference is simply to report the numbers receiving both IASI and KA within the results section, but not to redo all the analyses with this third exposure group. However, if the editors specifically request us to redo all analyses using this additional exposure group, we would be happy to do so.

5. **Whether it is possible to cite patients who have received both intraarticular steroid injection and arthroscopic knee surgery as a class, the relevant literature is as recent as possible**

To our knowledge there has been no prior research study that has investigated the impact of patients receiving both IASI and KA prior to knee replacement. Whilst investigating this exposure group was not a research aim of our present study, it could be an interesting topic for future research works.

6. **The good thing about this paper is the large amount of data, but it lacks innovation. Similar studies have been done in the past**

Whilst similar studies have been done in the past, the literature remains inconclusive. Our study is one of the largest looking into this important topic that has still to be conclusively answered. The topic is very relevant to orthopaedic surgeons, community GPs and of course patients that commonly undergo IASI and KA prior to TKR.

7. **Please explain why the mortality associated results were seen in the patients who had steroid injection and arthroscopy first mentioned in the paper, and what might be the confounding factors?**

See the earlier answer to Question 3 which further addresses the association seen with IASI and arthroscopy. We believe selection bias and younger age at Primary Knee Replacement is the key confounder by indication in the KA group and is accounting for the reduced mortality. We do not believe there is a biological cause for the reduction in mortality seen with prior IASI users.

**Reviewer 2:**

**The results of this observational analysis imply that poorer outcomes are linked to both knee arthroscopy and steroid injections before primary knee replacement.**

**Some consideration should be added to the discussion.**

We have summarised the key findings in the discussion section, where we observe that patients receiving IASI or KA prior to primary knee replacement have poorer outcomes. Specifically, that both IASI and KA groups both have higher rates of revision and re-operation. That patients receiving IASI take an average of 6-months longer to receive knee replacement, and KA being associated with lower clinically meaningful improvement in patients reported pain and function.

**Studies have shown that knee osteoarthritis can be effectively treated with injections of sodium hyaluronate and platelet-rich plasma (PRP); nevertheless, in certain cases, PRP may be more advantageous than arthroscopic debridement (10.21037/APM-21-2145). Despite their association with an increased risk of postoperative infection (10.7759/cureus.61649),. Moreover, the role of rehabilitation in those cases should be considered (10.3390/app132413208).**

The introduction has been updated to included further information on alternative treatments in OA and relevant citations included.

**The conclusion paragraph in the article should be different and more argumentative than in the abstract**

The conclusion paragraph has been amended. We are being cautions with our interpretation and conclusion from the study. Our study is observational data analysis and there is always potential for residual confounding even with robust statistical analysis, as we have described in our limitations section.
